# Interpericyte Tunneling Nanotubes Are Nonuniformly Distributed in the Human Macula

**DOI:** 10.1167/iovs.65.13.28

**Published:** 2024-11-14

**Authors:** Martin Hein, Hassanain Qambari, Paula Yu, Dao-Yi Yu, Chandrakumar Balaratnasingam

**Affiliations:** 1 Lions Eye Institute, Perth, Australia; 2 Centre for Ophthalmology and Visual Science, University of Western Australia, Perth, Australia; 3 Department of Ophthalmology, Sir Charles Gairdner Hospital, Perth, Australia

**Keywords:** retina, microvasculature, pericyte, nanotubes, perfusion

## Abstract

**Purpose:**

Pericyte-to-pericyte communication via interpericyte tunneling nanotubes (IP-TNTs) is an important mechanism by which spatial and temporal precision in neurovascular coupling is achieved. This study quantifies the distribution and morphologic characteristics of IP-TNTs in the normal human macula.

**Methods:**

Ultra high-resolution, three-dimensional microscopic imaging of 11 perfusion-labeled normal human donor eyes was performed. Immunofluorescent markers for collagen IV, glial fibrillary acidic protein, nuclei, α-smooth muscle actin and phalloidin were used to distinguish IP-TNTs from perfused/nonperfused capillaries and glia processes. IP-TNT length, diameter and density in each capillary plexus was quantified and compared.

**Results:**

IP-TNTs were present in all capillary plexuses. IP-TNTs bridged capillary segments within and between capillary plexuses but did not connect capillaries to arterioles or venules. Mean length of IP-TNTs was 72.6 ± 39.5µm (range 14.0 to 202 µm) and mean diameter was 1.0 ± 0.42µm. IP-TNT length was non-normally distributed with a right-skewed distribution and 43% were ‘short’ (<55µm). Diameters were greater in the “long” (1.13 ± 0.44 µm) than “short” (0.82 ± 0.33 µm; *P* < 0.001) IP-TNTs. Density of IP-TNTs was greater in the superficial vascular plexus (3.80 ± 0.69 per 500 µm^2^) compared to the intermediate (1.85 ± 0.80 per 500 µm^2^; *P* < 0. 0001) and deep capillary plexus (1.58 ± 0.84 per 500 µm^2^; *P* < 0.0001). No significant difference in IP-TNT density was found between the four macula quadrants (*P* = 0.98).

**Conclusions:**

The distribution of IP-TNTs in the human macula is non-uniform and is associated with the compartmentalized nature of retinal energy consumption and microvascular perfusion. The nonuniform properties of IP-TNTs may predispose distinct vascular beds to injury in conditions such as diabetes.

Retinal microvascular blood flow needs to be precisely fine-tuned to match the rapidly changing energy demands of retinal elements.^1^ Pericytes are strategically positioned within the retinal microcirculation and regulate blood flow by constricting and dilating capillary structures.^2^^–^^4^ There are immense spatial and temporal variations in retinal microvascular blood flow and pericyte-mediated neurovascular coupling is critical for ensuring blood flow increases within defined capillary networks while simultaneously reducing blood flow in others.^1^^,^^3^^,^^5^^,^^6^ Rapid cell-to-cell communication is a fundamental mechanism by which pericytes facilitate the spatial and temporal accuracy of microvascular blood flow regulation.^7^^,^^8^

Tunneling nanotubules (TNTs) are intercellular structures that allow immediate cell-to-cell communication through the selective transfer of membrane vesicles, calcium and mitochondria.^9^ TNTs have been identified in a range of central nervous system (CNS) cell types including astrocytes, neurons, microglia, and endothelia.^7^^,^^10^^–^^14^ Alarcon-Martinez et al.^7^ performed an elegant evaluation of interpericyte tunneling nanotubes (IP-TNTs) in the retina of mice that expressed fluorescent protein under the control of neural/glial antigen 2 (CSPG4) promoter and showed that IP-TNTs are essential for pericyte-to-pericyte communication and neurovascular coupling. That report together with a subsequent study from the same group showed that damage to IP-TNTs can impair blood flow regulation and neurovascular coupling in states of ischemia and intraocular pressure elevation.^7^^,^^15^

The human macula is a highly metabolically active retinal area responsible for high acuity vision and is characterized by a highly specialized microcirculation that is topologically different to nearly every other species.^16^ Microvascular perfusion abnormalities are a major pathogenic mechanism in human retinal vascular diseases,^17^^,^^18^ but there is very little information regarding the topologic distribution and quantitative characteristics of IP-TNTs in the human macular vasculature. The fovea of the macula provides a topographic landmark for replicable analysis of IP-TNTs. In this study we use perfusion labeling of human donor eyes to characterize and quantify IP-TNTs in the different capillary networks of the normal macula. The findings of this report improve our knowledge of retinal vascular physiology and can also be applied to expand our understanding of the pathogenic mechanisms underlying retinal vascular diseases such as diabetic retinopathy.

## Methods

### Human Donor Tissue

Human donor eyes were obtained from the Lions Eye Bank, Nedlands, Western Australia, and DonateLife, the organ and tissue retrieval authority in Western Australia. All human eyes used for this study were collected from patients with no documented history of ocular disease, diabetes mellitus or systemic vascular disease. The study was approved by the Human Research Ethics Committee at the University of Western Australia. Donor tissue was handled in accordance with the tenets of the Declaration of Helsinki.

#### Tissue Preparation and Cannulation

Human donor eyes were retrieved <18 hours postmortem and transported in ice-cold, carbogen-bubbled (95% O_2_, 5% CO_2_) sodium Ringer's solution and the cornea was removed for transplantation. We then cannulated the central retinal artery to deliver antibody directly to retinal microvasculature and the detailed methodology of that step is described in our previous publications.^19^^–^^22^ In brief, the central retinal artery is identified and dissected free from retro-orbital fat, and the eye is placed in a custom holder. The perfusion system is flushed with 0.1M phosphate buffer (PB) before cannulation to clear out debris and air bubbles that may occlude retinal vessels. Using a dissecting microscope, the central retinal artery is cannulated with a custom glass micropipette (100 µm tip size) and tied in place with an 11-0 suture. Successful vessel cannulation is confirmed by extrusion of blood from the central retinal vein when solutions are perfused.

#### Isolated Ocular Perfusion Labeling and Dissection of Human Donor Retinae

The glass micropipette cannulating the central retinal artery is attached to a custom cannula holder connected to a 10 mL syringe held in a syringe pump (Model 22; Harvard Apparatus Inc., MA, USA). The syringe pump provides a constant perfusion rate through the cannulated vessel. The cannulated eyes are perfused at a constant flow rate of 80 µL/min with the following solutions in order: washed with heparinized Ringer's solution for 30 minutes, fixed with −20°C 100% methanol for 20 minutes and permeabilized with 1% Triton-X 100 in 0.1M PB for 10 minutes. The use of methanol fixation and permeabilization with Triton-X 100 enables penetration of antibody beyond the vascular lumen to enable immunostaining of extraluminal structures. The following primary antibodies are then manually pushed (three boluses of 333 µL in 20-minute intervals): goat anti-collagen IV (NBP1-26549; Novus Biologicals, Littleton, CO, USA; 15 µL in 1000 µL 0.1 M PB), Hoechst 405 (H6024; Sigma-Aldrich, St. Louis, MO, USA; 2 µL in 1000 µL 0.1 M PB), mouse anti-⍺-smooth muscle actin (⍺SMA) (A2547; Sigma-Aldrich; 15 µL in 1000 µL 0.1 M PB), phalloidin (TRITC, P1951; Sigma-Aldrich, 1 µg in 1000 µL 0.1M PB) and donkey serum (100 µL in 1000 µL 0.1 M PB). Secondary fluorescent markers are then manually pushed at the same rate: donkey anti-goat Alex Fluor 488 (Ab150129; Abcam, Cambridge, MA, USA; 15 µL in 1000 µL 0.1 M PB). The central retinal artery is decannulated and the iris and lens is removed. Four or five radial cuts centering on the optic disc are made, full thickness, through the retina, choroid, and sclera. Each leaflet is fanned out to flatten the specimen and expose the vitreous humor that is subsequently removed. The retina is then peeled from the choroid, dissected at the optic disc and floated for 24 hours in rabbit anti-GFAP (G6171; Sigma-Aldrich; 20 µL in 1500 µL 0.1M PB). Secondary float is performed overnight with donkey anti-rabbit Alex Fluor 647 (Ab150075; Abcam, 20 µL in 1500 µL 0.1 M PB) and donkey anti-mouse Alex Fluor 555 (Ab150111; Abcam; 15 µL in 1000 µL 0.1M PB). After a final wash in 0.1 M PB, the entire retina is whole mounted in glycerol for confocal microscopy.

### Image Acquisition

Retinal flat mounts are imaged using the Nikon C1 plus confocal system (Nikon Corporation, Tokyo, Japan) equipped with four solid-state lasers at wavelengths 405 nm, 488 nm, 561 nm, and 635 nm. Macular areas for density calculations are imaged within a 5.5mm diameter circular area centered on the fovea using a Nikon Plan Apochromat VC 20x (NA 0.75) dry objective lens. Each macular area is optically sectioned to accurately distinguish different vascular layers using co-localization of nuclei stained in each retinal layer. Using definitions from our previous reports, vessels of the superficial vascular plexus (SVP) were located in the nerve fiber layer (NFL) and retinal ganglion cell layer (GCL).^16^^,^^23^ The intermediate capillary plexus (ICP) vessels are located in the deep portion of the inner plexiform layer and superficial portion of the inner nuclear layer and deep capillary plexus (DCP) vessels are located within the deep portion of the inner nuclear layer and the outer plexiform layer.^16^^,^^23^ High magnification images were acquired using a Nikon 60x (NA 1.40) oil objective lens and 100x (NA 1.40) oil objective lens.

### Histologic Criteria For Identification of Interpericyte Tunneling Nanotubes (IP-TNTs)

**Figure 1.**
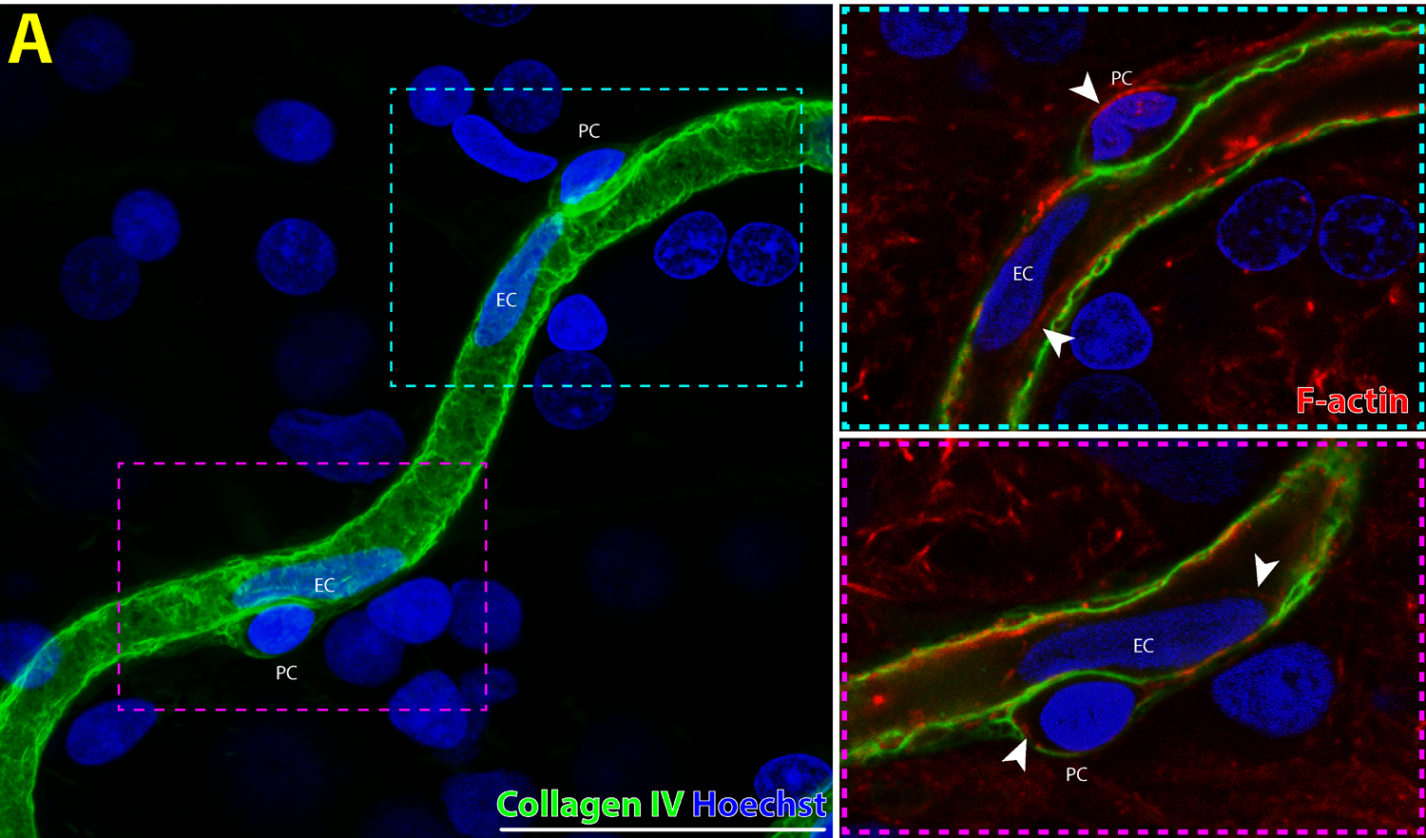
Endothelial (EC) and pericyte (PC) morphology seen on a single perfused retinal capillary in the normal human macula (**A**). PCs are cells with dome-shaped nuclei that are found on the abluminal surface of retinal microvasculature. Capillary ECs have elongated nuclei that are oriented parallel to blood flow in perfused capillaries. Both pericytes and endothelial cells contain intracellular filamentous actin (F-actin; red stain) cytoskeletal fibers that are visible in single optical sections of the same scanned area (*white arrowheads*, *cyan* and *magenta inset*). *Scale bar*: 50 µm. *Green* = collagen IV; *Blue* = Hoechst; *Red* = F-actin.

**Figure 2.**
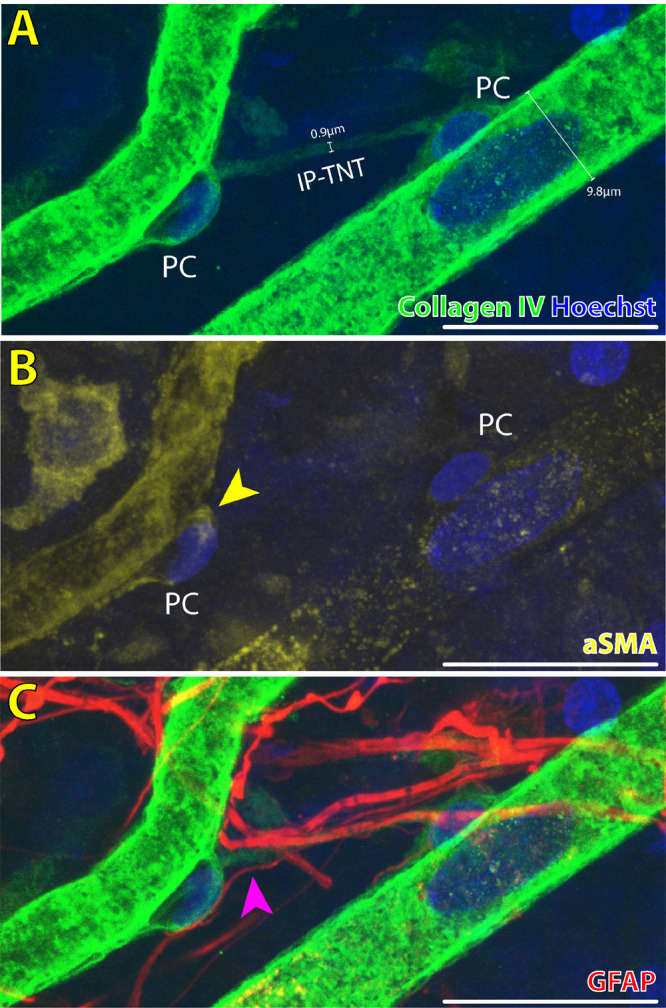
Immunofluorescent staining characteristics of IP-TNTs in the superficial vascular plexus of normal human retinal vasculature. IP-TNT connecting two pericytes (PC) on adjacent capillary segments stains positive for basement membrane antibody collagen IV (**A**). The IP-TNT is significantly narrower in diameter (0.9 µm) than either adjacent capillary and no PCs or endothelial cells are visible along the length of the IP-TNT. PCs imaged here are positive for αSMA (**B**; *yellow arrowhead*), but the bridging IP-TNT does not demonstrate positive αSMA immunolabeling. GFAP-positive astrocyte processes intimately envelope the IP-TNT (**C**), but the IP-TNT itself is a distinct structure from GFAP-positive astrocyte processes, which are significantly more numerous than IP-TNTs (**C**; *magenta arrowhead*). *Scale bars*: 20 µm. *Green* = collagen IV; *Blue* = Hoechst; *Red* = GFAP; *Yellow* = αSMA.

**Figure 3.**
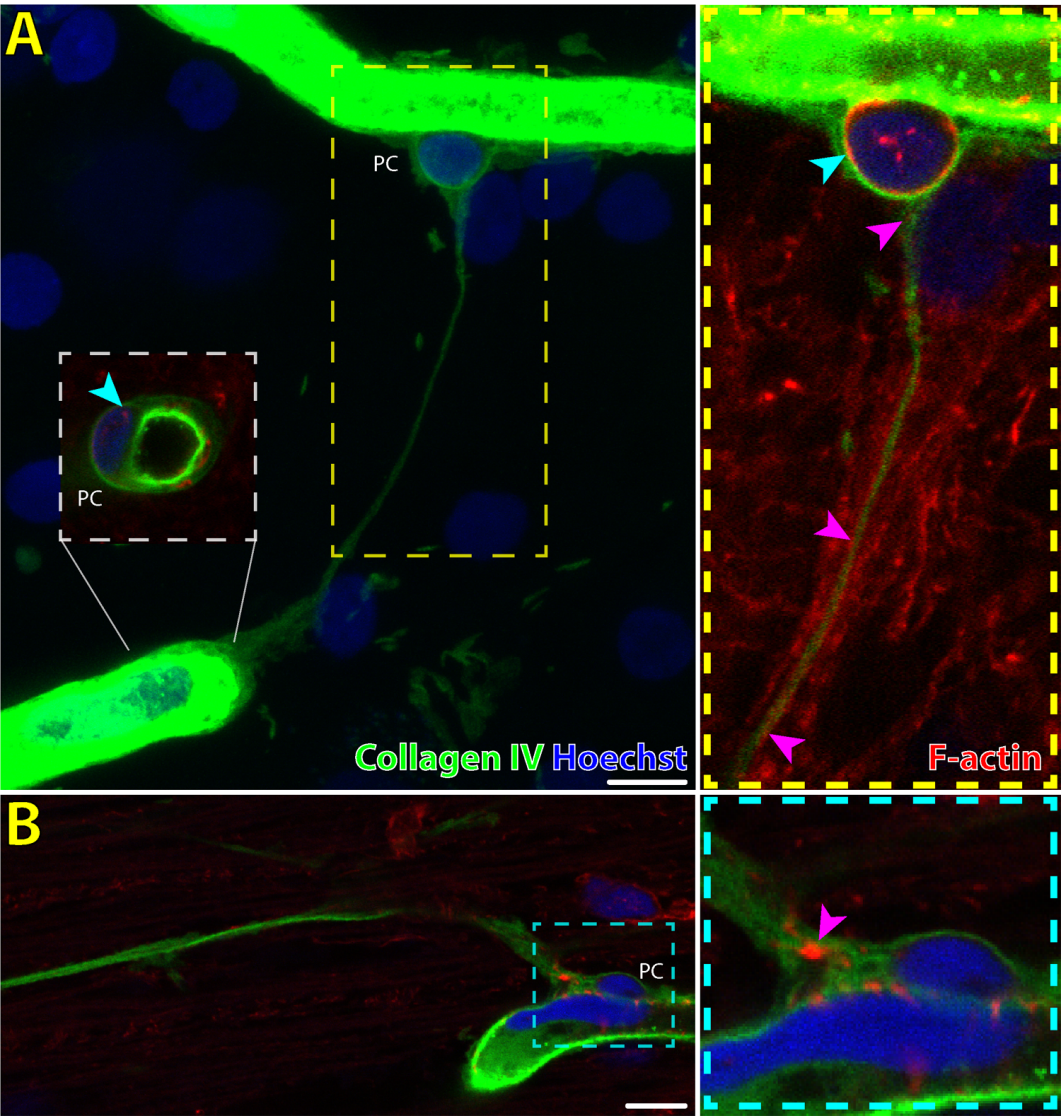
Immunofluorescent labeling of IP-TNT and pericytes in normal human retina. An IP-TNT is shown bridging between two pericytes (PC) on adjacent capillary segments (**A**). The IP-TNT is positive for basement membrane antibody collagen IV and a single optical section of the same IP-TNT (*yellow dashed inset*) reveals subtle expression of filamentous actin (F-actin; *magenta arrowheads*) along the course of the IP-TNT. Both pericytes of the IP-TNT in (**A**) exhibit F-actin expression within their cell bodies (*cyan arrowheads*). (**B**) A different IP-TNT (second pericyte not imaged) with more obvious F-actin expression (*cyan inset; magenta arrowhead*). *Scale bars*: 10 µm. *Green* = collagen IV; *Blue* = Hoechst; *Red* = F-actin.

**Figure 4.**
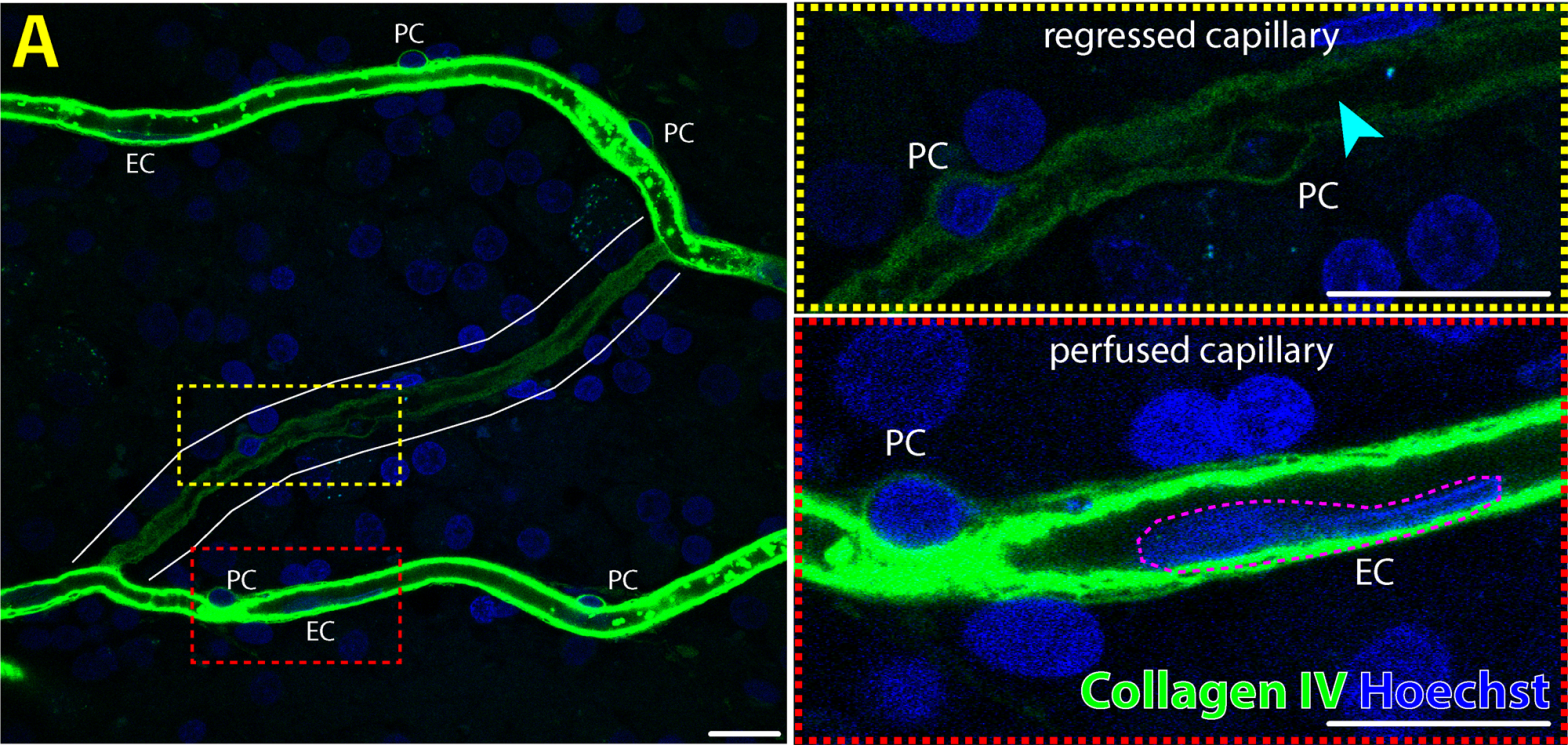
Immunofluorescent labeling of perfused and regressed/non-perfused capillaries in the normal human retina with collagen IV and nuclei marker. A regressed capillary segment (*white solid outline*) is visible bridging between two perfused capillaries (**A**). Pericytes (PC) and a residual lumen (*cyan arrowhead*) are visible along the regressed capillary, which stains weakly for basement membrane marker, collagen IV (*yellow dashed inset*). No endothelial cell (EC) nuclei are visible along the length of the regressed capillary. PCs and EC nuclei (*magenta dashed outline*) are visible along on the perfused capillary segments (*red dashed inset*). *Scale bar*: 15 µm. *Green* = collagen IV; *Blue* = Hoechst.

**Figure 5.**
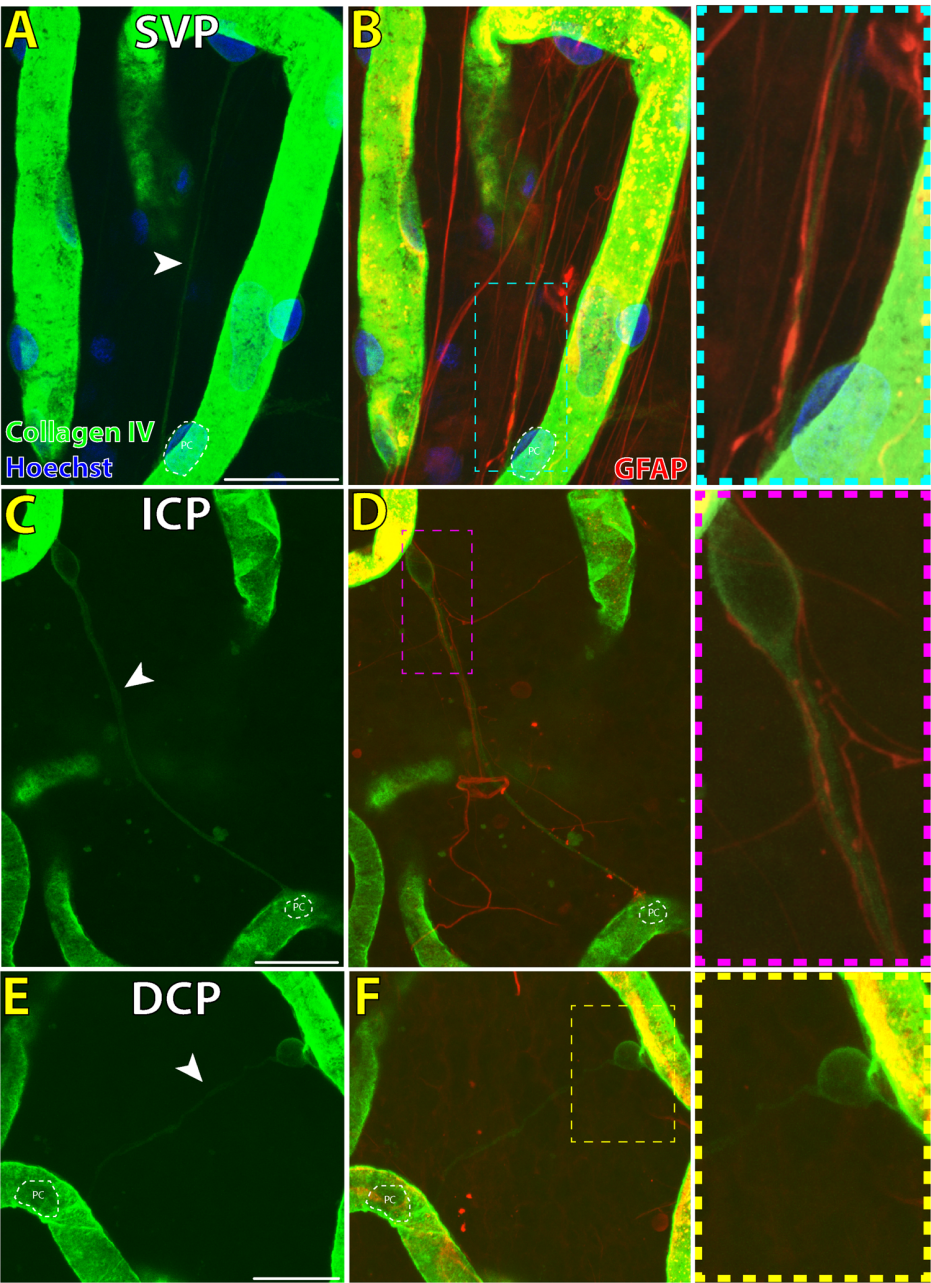
IP-TNTs and retinal glia immunolabeling across different vascular layers in normal human retinal vasculature. IP-TNTs that are narrow collagen IV positive structures (*white arrowheads*) bridging between two pericytes (PC) are seen in all vascular layers, the superficial vascular plexus (SVP) (**A**, **B**), intermediate capillary plexus (ICP; **C**, **D**) and DCP (**E**, **F**). In the SVP and ICP, GFAP-positive processes of astrocytes intimately envelope IP-TNTs (**B**, *cyan inset*; **D**, *magenta inset*). In the DCP, GFAP positive retinal glia processes are less frequently seen (**F**, *yellow inset*). Two examples of IP-TNTs with PCs “detached” from the parent capillary are seen here (**D**, **F**); this morphology is also seen in human CNS IP-TNTs. **Panel A** shows an IP-TNT that has connected two PCs on the same capillary segment. *Scale bars*: 20 µm. *Green* = collagen IV; *Blue* = Hoechst; *Red* = GFAP.

Histologic criteria for defining the elements of the retinal vasculature were as follows:

1. Pericytes are abluminal cells with dome shaped nuclei that partially ensheathe capillaries (Fig. 1).^19^ Our previous studies found the combination of nuclei, ⍺SMA and filamentous actin (F-actin) labeling with phalloidin was sufficient for accurate identification of pericytes (Figs. 1–3).^16^^,^^24^^,^^25^ Because of nonspecific binding of neural/glial antigen 2 (NG2) antibody in our human retinal specimens, use of this antibody for identification of pericytes was not included.
2. Endothelia line the intraluminal surface of retinal capillaries and were identified according to their elongated nuclei shape oriented parallel to blood flow and presence of intracellular F-actin stress fibers (Fig. 1).^19^
3. Perfused retinal capillary segments were defined as per our previous reports (Figs. 1 and 4)^16^^,^^26^:
  • Diameter less than 10 µm
  • Single layer of nucleated endothelial cells forming the capillaries’ tubular structure
  • Pericytes may be present along the length of the capillary
  • Stained positive for collagen IV
4. Regressed or non-perfused retinal capillary segments, also referred to as “ghost vessels” (Fig. 4)^27^:
  • Residual basement membrane shell staining weakly for collagen IV
  • May demonstrate pericytes or denucleated pericyte shells
  • No endothelial cell nuclei present along the length of the vessel segment
  • A residual lumen is occasionally visible
5. Astrocyte processes^22^ (Fig. 2 & Supplementary Fig. S1):
  • Positive staining for glial fibrillary acidic protein (GFAP)
  • Cell bodies and processes present in NFL and GCL with some processes extending deeper into the inner plexiform layer and inner nuclear layer^22^
  • Astrocyte processes take on a stellate morphology that intercalate with retinal vessels or an elongated morphology that run parallel with nerve fibers in the NFL^22^
6. IP-TNTs^10^^,^^28^:
  • Mean luminal diameter of approximately 0.5-1 µm as per previous histologic measurements of IP-TNT in human CNS and rodent retina (Fig. 2) ^7^^,^^11^
  • A non-branching tubule that connects two pericytes
  • Negative staining for GFAP (Figs. 2, 5)
  • Did not contain pericytes or endothelial cells along the length of the tubule as would be seen in perfused and regressed capillaries (Figs. 1, 4)
  • Stained positive for collagen IV (Figs. 2, 3)^7^^,^^11^

### Quantitative Analysis of IP-TNT in the Macula

The density of IP-TNTs in each vascular plexus (SVP, ICP, DCP) were calculated using manual counting of IP-TNTs across 500 µm^2^ retinal areas at four regions (superior, inferior, temporal, nasal) surrounding the foveal avascular zone, prior to vascular layer convergence towards the terminal capillary ring and within the 5.5 mm diameter macular area of each donor eye (Supplementary Fig. S2). The diameter and length of identified IP-TNTs are measured at three equidistant points along their course using high magnification scans and three-dimensional image stack reconstructions with NIS-Elements AR package (version 5.30.07). Statistical analysis between density counts of IP-TNTs in different vascular layers and between diameters of IP-TNTs and regressed capillaries was performed using two-tailed Mann-Whitney U test for nonparametric data. Analysis of IP-TNT density counts between the four quadrants was performed using Kruskal-Wallis test for non-parametric data. Results presented as mean ± standard deviation.

## Results

Eleven donor eyes from eight human donors were used in this study. The Table summarizes donor characteristics.

**Table.** Human Donor Details

Donor (No. of Eyes)	Age (years)	Sex	Postmortem to Fixation Time	Cause of Death
1 (2 eyes)	63	M	18 hours	Metastatic prostate cancer
2 (1 eye)	65	F	17hours	Intracranial hemorrhage
3 (2 eyes)	62	F	17 hours	Decompensated liver disease
4 (1 eye)	67	M	11 hours	Intracranial hemorrhage
5 (2 eyes)	64	M	7hours	Motor neuron disease
6 (1 eye)	70	M	12hours	Metastatic hepatocellular carcinoma
7 (1 eye)	68	M	12 hours	Decompensated liver disease
8 (1 eye)	61	M	16 hours	Intracranial hemorrhage

### Qualitative Characteristics of IP-TNTs in the Macula

**Figure 6.**
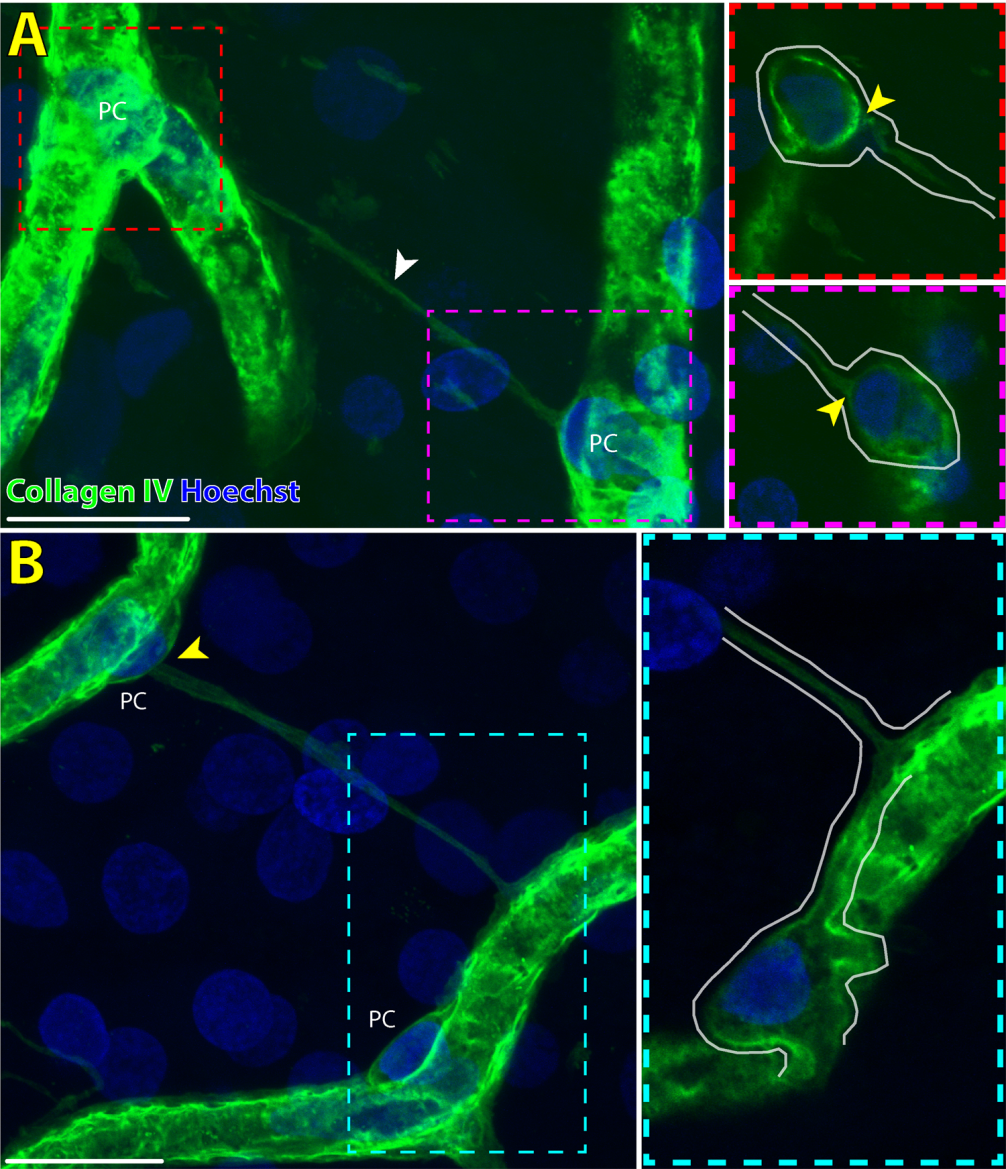
High-magnification images of two IP-TNTs interfacing with pericytes (PC) on adjacent capillary segments in the normal human macula. (**A**) The IP-TNT (*white arrowhead*) interfaces directly with the cell body of both pericytes (*yellow arrowheads*). The two pericytes are shown in single optical sections of the same scan (*white outline, red* and *magenta inset*). (**B**) Different capillary segments, the IP-TNT extends from the cell body of a single pericyte (*yellow arrowhead*) and interfaces with pericyte processes distal from the second pericyte cell body, which extend toward the IP-TNT (*cyan inset*). The approximate shape of the pericytes processes is demarcated (*cyan inset, white outline*). *Scale bars*: 20 µm. *Green* = collagen IV; *Blue* = Hoechst.

Thin collagen IV positive tubules connecting two pericytes that were morphologically similar to what has been described in the human brain and rodent retina were seen in all vascular plexuses of the human macula (Supplementary Fig. S3).^7^^,^^11^ The thin tubules of IP-TNTs were seen to either directly interface with pericyte cell bodies or with pericyte processes distal to the pericyte cell body (Fig. 6, Supplementary Fig. S4). The bridging tubules of IP-TNTs were observed to be closely intertwined with GFAP positive astrocyte processes in the SVP and ICP (^1^Figs. 2, 5). In the DCP, IP-TNTs were less frequently seen to be associated with GFAP positive glia than the SVP or ICP (Fig. 5). IP-TNTs were seen to bridge between pericytes located on different vascular plexuses, from SVP to ICP, ICP to DCP and SVP to DCP (Supplementary Video S1). Occasionally “detachment” of a pericyte from the parent capillary onto the IP-TNT was also seen (Fig. 5C). A similar morphology has been documented in human CNS TNTs (Supplementary Fig. S3).^11^ IP-TNTs were not noted to bridge between small arterioles or venules but only between adjacent capillary segments or, less commonly, the same capillary segment (Fig. 5A). None of the IP-TNTs themselves expressed ⍺SMA positive filaments but were seen to connect with both ⍺SMA negative and positive pericytes (Fig. 2B). IP-TNTs and their pericytes demonstrate expression of F-actin within pericyte cell bodies and along the length of IP-TNTs (Fig. 3^4^,^5^,^6^). We also found that seven of the 72 (9.7%) IP-TNTs studied under high magnification contained a break along the length of the tubule, similar to what has been described in the rodent retinae and human CNS (Supplementary Fig. S3B), although this may be due to postmortem times and sample preparation.^7^^,^^11^^,^^29^

### Quantitative Characteristics of IP-TNTs in the Macula

**Figure 7.**
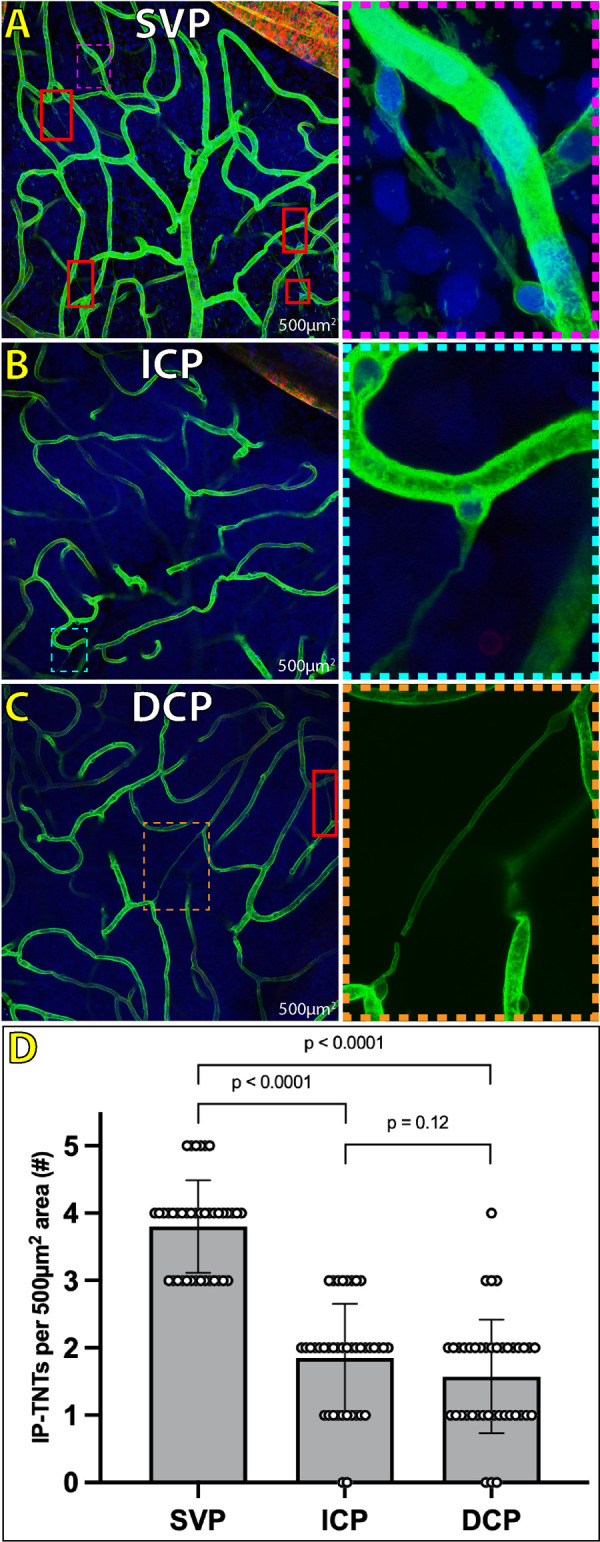
Density of IP-TNTs per vascular plexus in the normal human retina as measured in an area of 500 µm^2^ in the macula. IP-TNTs are present in every vascular plexus, SVP (**A**), ICP (**B**), and DCP (**C**) of the same macular area shown here. The SVP demonstrates a significantly greater density of IP-TNTs per 500 µm^2^ area in the human macula compared to the ICP and DCP (**D**; *P* < 0.0001). There is no significant difference between density of IP-TNTs in the ICP or DCP (**D**; *P* = 0.12). **Panel A** shows five IP-TNTs total in the SVP (*red outline*) with one magnified IP-TNT (*magenta inset*). The ICP and DCP of the same 500 µm^2^ macular area show significantly less IP-TNTs (**B**: *cyan inset*; **C:** *orange inset*). *Green* = collagen IV; *Blue* = Hoechst.

**Figure 8.**
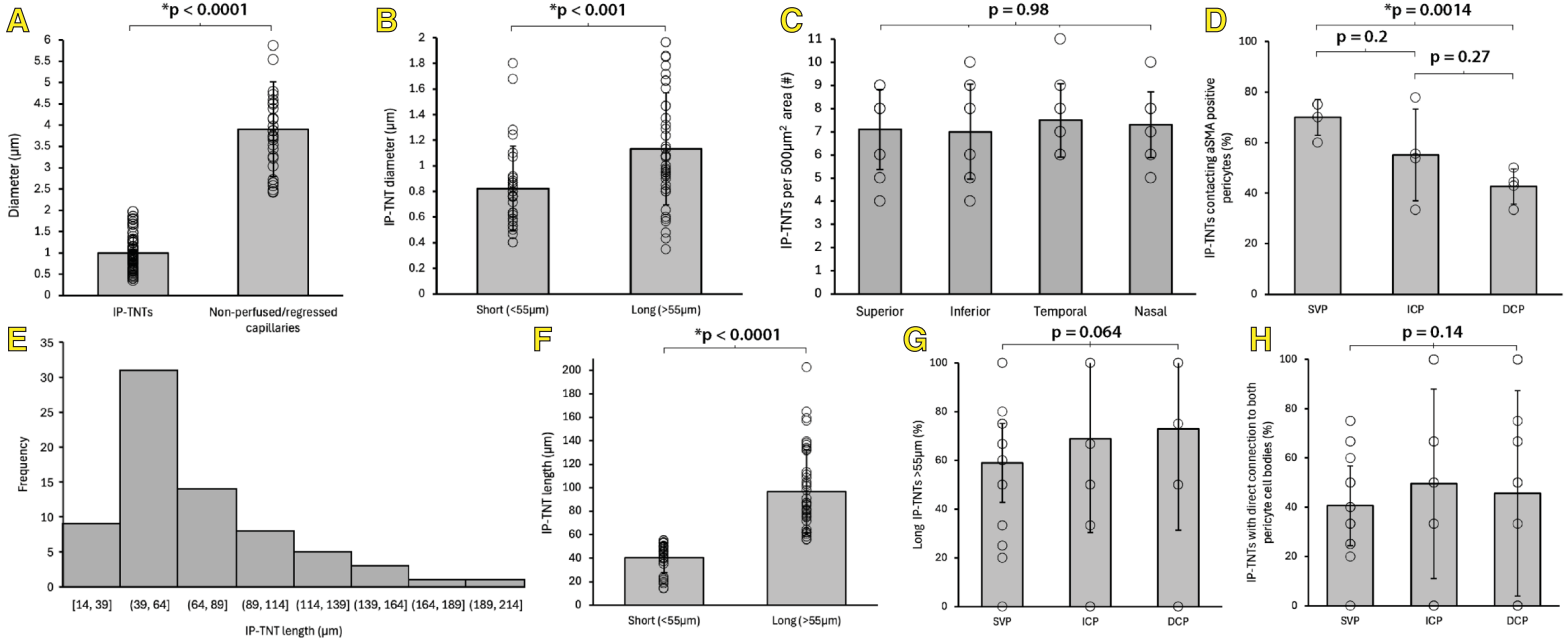
Quantitative characteristics of IP-TNTs in the macula of human retinae. (**A**) Diameter of IP-TNTs (*n* = 72) and nonperfused capillaries (*n* = 34). (**B**) Diameter between short (≤55 µm, *n* = 31) and long (>55 µm, *n* = 41) IP-TNTs. (**C**) IP-TNT density per macular quadrant. (**D**) Percentage of IP-TNTs in contact with αSMA per vascular plexus, SVP, ICP, and DCP (*n* = 94). (**E**) Right-skewed distribution of IP-TNT length. (**F**) Length of short and long IP-TNTs. (**G**) Percentage of “long” IP-TNTs per vascular plexus (*n* = 289). (**H**) Percentage of IP-TNTs that connect directly with both pericyte cell bodies per vascular plexus (*n* = 289). Data presented as mean ± standard deviation. * Denotes statistical significance.

Density calculations identified 289 IP-TNTs over a total macula area of 20,000 µm^2^ from 10 eyes (a single retina was excluded from density calculations because of artefactual distortion of vascular layers). The SVP had a significantly greater density of IP-TNTs (3.80 ± 0.69 per 500 µm^2^) compared to the ICP (1.85 ± 0.80 per 500 µm^2^; *P* < 0. 0001) and DCP (1.58 ± 0.84 per 500 µm^2^) (*P* < 0.0001) (Fig. 7, Supplementary Fig. S5). There was no difference in the density of IP-TNTs between the ICP and DCP (*P* = 0.12) (Fig. 7). IP-TNT density counts of the four macula quadrants showed no significant difference (superior quadrant = 7.1 ± 1.73 per 500 µm^2^) (inferior quadrant = 7.0 ± 2.05 per 500 µm^2^) (temporal quadrant = 7.5 ± 1.58 per 500 µm^2^) (nasal quadrant = 7.3 ± 1.42 per 500 µm^2^) (*H*(3) = 0.17, *P* = 0.98) (Fig. 8).

High magnification study of 72 IP-TNTs from 11 human retinae was performed. The interpericyte length of IP-TNTs ranged from 14.0 to 202 µm with a mean length of 72.6 ± 39.5 µm and a mean diameter of 1.0 ± 0.42 µm. The length of IP-TNTs studied here were non-normally distributed with a right-skewed distribution (Fig. 8). Alarcon-Martinez and colleagues^7^ described a bimodal distribution of length measurements in murine retinal IP-TNTs by choosing a 30 µm cutoff point for short and long IP-TNTs. By applying the same proportional cut off point (55µm) for the length range of human retinal IP-TNTs, we found 43% were short (≤55 µm, 40.5 ± 12.9 µm) and 57% of IP-TNTs were long (>55 µm, 96.8 ± 35.4 µm) (Fig. 8). The IP-TNT diameters were significantly greater in the long IP-TNTs (1.13 ± 0.44 µm) than short IP-TNTs (0.82 ± 0.33 µm) (*P* < 0.001) (Fig. 8). Proportion of long to short IP-TNTs did not differ between vascular plexuses (H(2) = 5.50, *P* = 0.064) (Fig. 8) or macular quadrants (H(3) = 1.59, *P* = 0.66). IP-TNTs in contact with at least one ⍺SMA positive pericyte was greatest in the SVP (70%) than ICP (55.1%) (*P* = 0.2) and significantly greater than in the DCP (42.7%) (*P* = 0.0014) (Fig. 8, Supplementary Fig. S6). Proportion of IP-TNTs that interface with distal pericyte processes was 54.8% and did not differ between vascular plexuses (H(2) = 1.45, *P* = 0.48) (Fig. 8) or macular quadrants (H(3) = 5.55, *P* = 0.14). The diameter of regressed capillaries (3.91 ± 1.11µm, *n* = 34) was significantly greater than structures identified to be IP-TNTs (1.0 ± 0.42 µm) (*P* < 0.0001) (Fig. 8).

## Discussion

In the brain, there is approximately 80% coverage of capillaries by pericytes.^30^ In the normal human retina, the ratio of pericytes to endothelia is 1:1 and both cells are embedded in the same basement membrane.^31^ These lines of evidence suggest that pericytes are possibly the most important cell type of the neurovascular unit that regulates microvascular perfusion. The abluminal position of pericytes relative to endothelia and the expression of contractile proteins such as ⍺SMA permit pericytes to precisely regulate capillary blood flow.^3^^,^^19^ Foundational work out of the laboratory of Attwell et al.^3^ showed in vivo that sensory-stimulated increases in blood flow in the CNS is predominantly regulated by capillary pericytes rather than arteriole smooth muscle cells. Despite firm evidence that pericytes play a vital role in tissue homeostasis, the mechanisms by which they control spatial and temporal changes in microvascular perfusion to meet heterogeneous metabolic demands of neuronal function are unclear.^1^^,^^5^

The topologic organization of pericytes is akin to a syncytium and intercellular signaling mechanisms between pericytes through the pericyte connectome may be one important way by which spatial and temporal precision of neurovascular coupling is achieved.^8^ Rustom and colleagues^9^ were one of the earliest investigators to describe long plasma membrane ridges that connected cells and facilitated cell-to-cell communications. These structures were observed in cultured rat pheochromocytoma cells and were referred to as “tunneling nanotubes”. Since that report, TNTs have been observed in a range of CNS cell types including brain endothelia, glia, neurons and pericytes.^7^^,^^10^^–^^14^ TNTs are cellular specializations that facilitate rapid intercellular communications within the CNS. Mechanisms by which TNTs facilitate rapid cell-to-cell communication include the transfer of small molecules such as calcium ions, macromolecules such as proteins and nucleic acid and entire organelles such as mitochondria and lysosomes.^32^

Few studies have examined the occurrence and role of TNTs in the retina. The seminal study undertaken by Alarcon-Martinez et al.^7^ on murine retina demonstrated that TNTs connect retinal pericytes and play a vital role in controlling neurovascular coupling. Imaging of retinal pericytes by two photon scanning laser microscopy in mice expressing GCaMP6, under the control of the neural/glial antigen 2 promoter revealed that interpericyte communication is achieved through bidirectional propagation of intercellular calcium waves. The authors also showed that laser-induced ablation of individual IP-TNTs severely compromised the ability of capillaries to control blood flow in response to neuronal activity.^7^ We found that IP-TNTs in the human macula shared several similarities to IP-TNTs reported in other organs and species.^7^^,^^11^ Notably, IP-TNTs were found to communicate directly with pericyte cell bodies, as well as with pericyte processes distal to the cell bodies. The observation suggests that open-ended cellular channels may exist when TNTs interface directly with pericyte cell bodies and closed-ended connections may occur from the junction of TNT end-feet with distal pericyte processes, as described in rodent retina.^7^^,^^10^ The length of IP-TNTs in the human macula ranged from 14.0 to 202 µm and is significantly greater than the mouse retina (4 to 90 µm) where a bimodal distribution of IP-TNT length was found. In humans we see a right-skewed distribution of TNT length with long IP-TNTs (57%), demonstrating significantly greater diameter, than short IP-TNTs, based on a cut off proportional to rodent retina definitions. This is different to the findings in murine retina where the majority are classified as short (66%), based on a 30 µm cut off, with no diameter difference. The disparity may be due to the greater lengths required by IP-TNTs to tunnel through the human retinal stroma and the greater widths required to structurally support the longer bridging tubules.

The mean length of IP-TNTs in the human macula was comparable to what has been reported connecting astrocytes and urothelial cells.^33^^,^^34^ Veranic and colleagues^34^ classified nanotubules of RT4 and T24 urothelial cell lines according to length and function. They reported that type 1 nanotubes are less than 30µm in length and typically establish a cytosolic rather than membrane connection between 2 cells. Type 1 nanotubules are dynamic due to the presence of actin and are capable of exploring their external surroundings. They typically appear as bunches of tubes that dynamically seek connections with neighboring cells. In contrast, type 2 nanotubes are longer than type 1 nanotubes and are predominantly located on the apical aspect of the cell. Type 2 nanotubes were also noted to form when two cells that are already connected start to grow apart. Retinal pericytes are dynamic structures that are mobile and capable of migrating away from areas of hypoxia.^35^^,^^36^ The distribution of IP-TNTs in the macula, with respect to length, may therefore confer important information regarding the stationary and motility characteristics of pericytes. IP-TNTs may operate as shortcuts between capillaries for migrating pericytes or represent a persistent intercellular connection that originally formed between spatially intimate pericytes that have since migrated to distant capillaries.

Retinal blood flow is markedly heterogeneous, and there are significant differences in rates of blood flow between different capillary networks.^1^^,^^5^^,^^6^ We recently performed a live, quantitative study of red blood cell movement in rodent retinal networks and found that blood flow speed in the superficial vascular layer (estimated marginal mean of 2405 ± 238.2 µm/s) was almost 1.5 times that of the deep vascular layer (1641 ± 173.0 µm/s) when breathing room air.^5^ In the present study we showed that the density of IP-TNTs was significantly greater in the SVP than the ICP or DCP. There might be several reasons to account for this finding: (1) Capillaries of the SVP receive disproportionately greater macula arteriolar inflow, and IP-TNTs may play a critical role for the downstream distribution of blood to the ICP and the DCP.^16^ The connection of IP-TNTs between capillary plexuses in the human macula as we have seen here may provide a feedback mechanism for the bidirectional control of blood flow between capillary systems. (2) There is greater fluctuation of energy demands for visual function within the NFL and GCL of the retina and IP-TNTs play a crucial role in fine tuning blood flow to match the relatively greater fluctuations in energy requirements. In vivo studies show that the inner retina, where the NFL and GCL reside, have a significantly greater consumption of oxygen than outer retinal layers.^37^^,^^38^ (3) A proportion of tissue oxygenation requirements at the level of the DCP may be derived from the choroidal circulation thereby reducing the need for specializations such as IP-TNTs in this layer.^39^^–^^41^ (4) The significantly reduced expression of ⍺SMA in the DCP (Supplementary Fig. S6) suggests a lower potential for blood flow fluctuations in this layer and a lesser requirement for IP-TNTs.^25^ In addition to the mean speeds of red blood cell movement being different between the superficial and deep vascular layers, our in vivo study also revealed that the speed of red blood cell movement was not uniform within capillary segments of each layer.^5^ The specialization of the primate macula in terms of vascular organization, neuronal density, and high metabolic activity of inner retinal layers may explain the greater density of IP-TNTs in the superficial vascular layers when compared to murine models.^16^^,^^37^^,^^38^^,^^42^ In the current study, we found evidence of TNTs connecting pericytes within the same capillary segment, and many of these IP-TNTs were enveloped by GFAP positive glia processes. Being embedded in the neurovascular unit, IP-TNTs may contribute to the control of local blood flow and modulate oxygen unloading by red blood cells in response to changes in neuronal energy requirements.

A major strength of this study is the application of ultra-high-resolution, three-dimensional microscopic imaging of perfusion-labeled donor eyes together with established histologic criteria to define IP-TNTs. Our well-established quantitative methodology, together with a range of immunohistochemical markers, allowed us to distinguish IP-TNTs from regressed capillaries and glia processes. The limitation of this study is the restricted sample size and the absence of dynamic data to investigate the role of IP-TNTs in functional hyperemia in humans. The challenge of postmortem to fixation times with human donor tissue may affect the integrity of finer IP-TNTs and contribute to the lower total density counts in human samples compared with rodent specimens. Important future directions of this research will be to investigate the pathogenic role of IP-TNTs in human retinal vascular diseases such as diabetes. It is possible that injury or loss of IP-TNTs is an early biomarker of diabetic retina disease and accounts for some of the very earliest clinical manifestations prior to retinopathy, such as an increase in capillary diameter and retinal blood flow.^18^^,^^26^^,^^43^ The reduced number of IP-TNTs in the DCP as seen in this study may also help explain the vulnerability of the DCP to vascular injury in diabetes.^25^^,^^26^^,^^44^^,^^45^ Alarcon-Martinez and colleagues^7^^,^^15^ demonstrated that damage and dysfunction of IP-TNTs as evidenced by loss of pericyte-to-pericyte communication and impaired light-evoked neurovascular coupling plays an important pathogenic role in glaucoma. Similar pathogenic mechanisms may underlie diabetic retinopathy and retinal vein occlusion.

## Supplementary Material

Supplement 1

Supplement 2

## Supplementary Material

**Supplementary Video**. Interpericyte tunneling nanotube (IP-TNT, white arrowhead) in the normal human macula bridging between two pericytes (PC) located on capillaries of the superficial vascular plexus (SVP) and deep capillary plexus (DCP), bypassing the intermediate capillary plexus (ICP). Green = collagen IV; Blue = Hoechst.
