# Supplementary material for: Interpericyte Tunneling Nanotubes Are Nonuniformly Distributed in the Human Macula
Source: Invest Ophthalmol Vis Sci. 2024 Nov 14;65(13):28. doi: 10.1167/iovs.65.13.28 (PMC11572754; doi:10.1167/iovs.65.13.28)
Supplement: Supplement 1 [file iovs-65-13-28_s001.pdf]

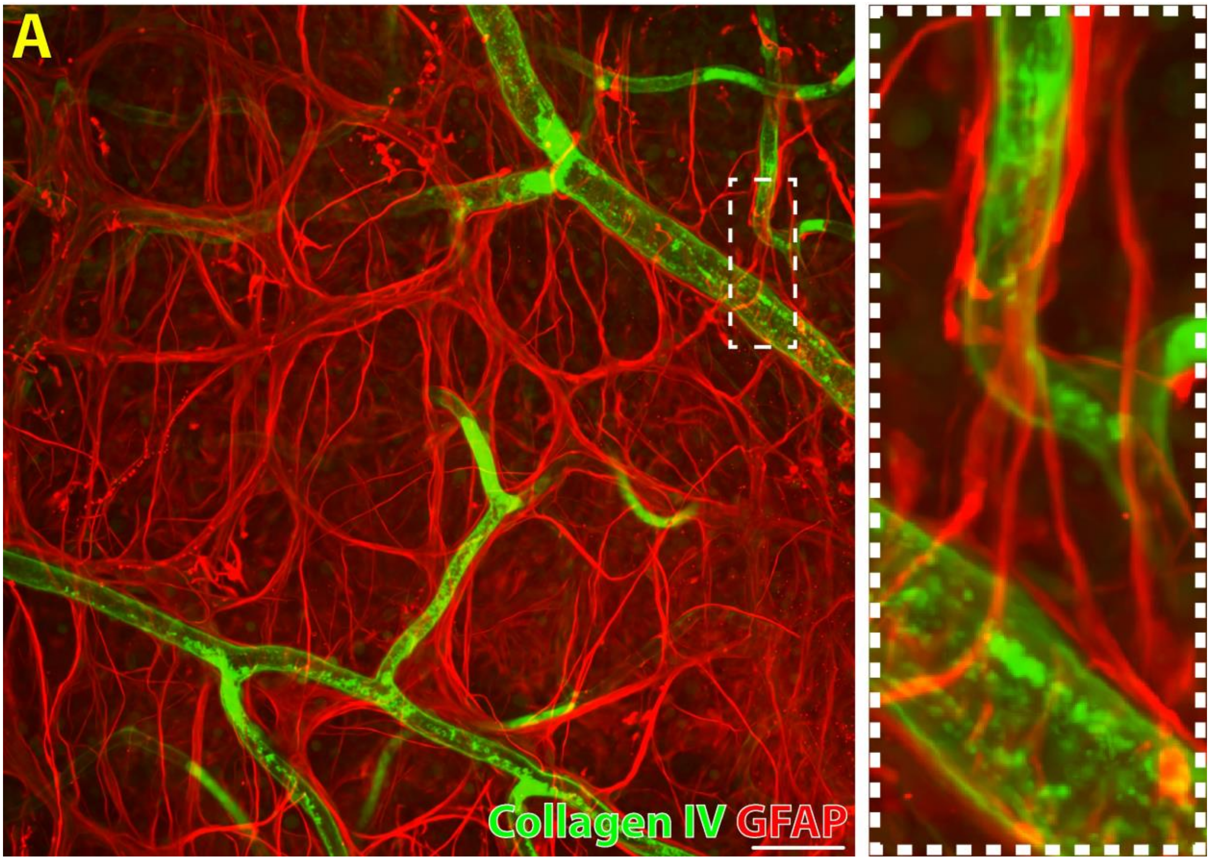

Supplementary Figure 1. Retinal astrocyte and astrocyte process morphology in the superficial vascular plexus of normal human macula vasculature. Astrocytes are retinal glia that stain positive for glial fibrillary acidic protein (GFAP) and take on a stellate morphology (A) with processes that intercalate with retinal vessels and capillaries (white dashed inset), or a longitudinal morphology with processes that run parallel with the nerve fibre layer (not pictured here). Scale bar = 50μm. Green = collagen IV; Red = GFAP.

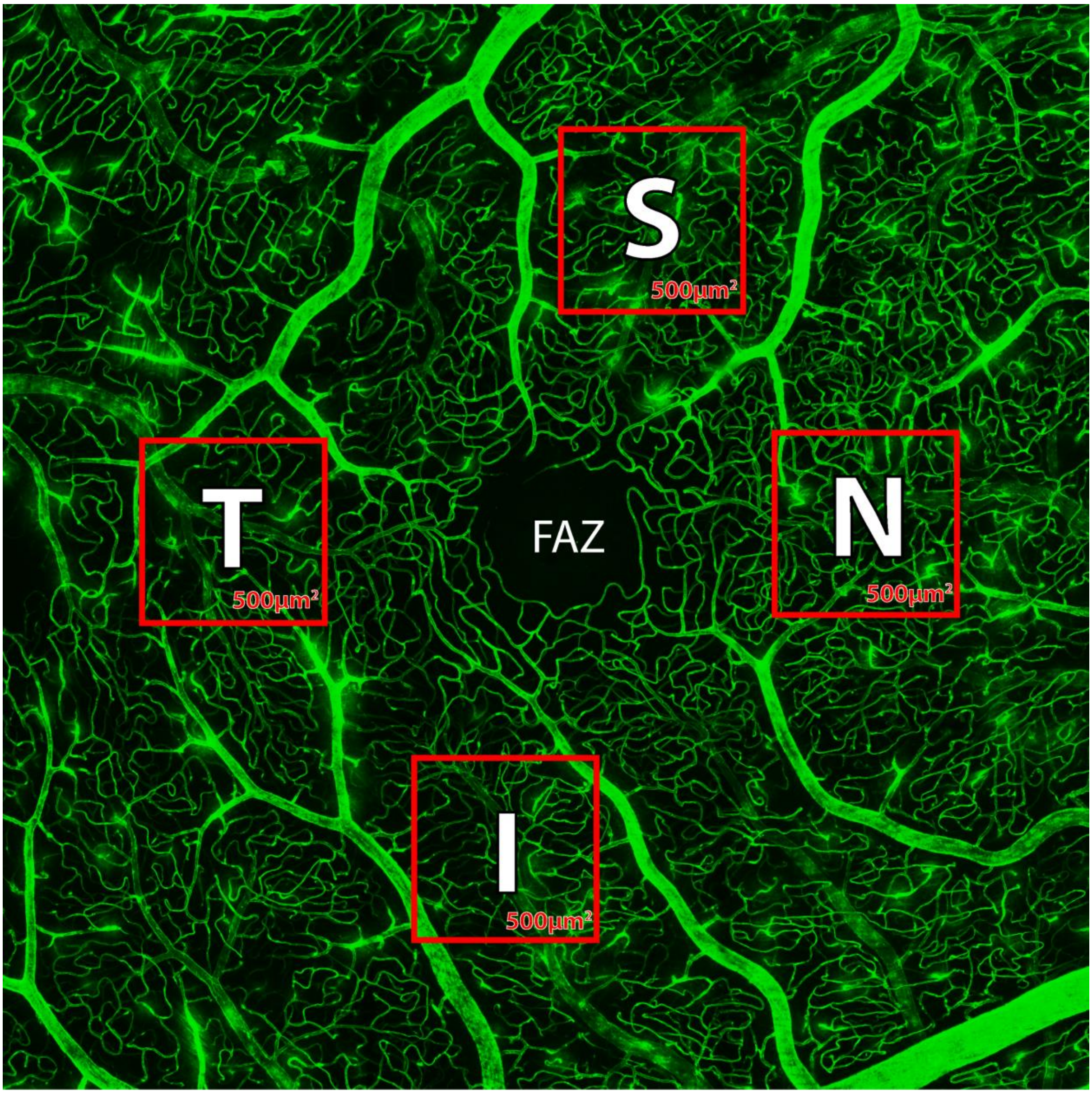

Supplementary Figure 2. Example depiction of four 500µm^2 areas for interpericyte tunnelling nanotube (IP-TNT) density analysis in human donor macula. Each area surrounds the foveal avascular zone (FAZ) and is superior (S), inferior (I), nasal (N) and temporal (T) in each specimen. In each area, IP-TNTs are counted from the superficial, intermediate and deep plexus. Care is taken to select a capillary dominant area for IP-TNT counting as to avoid artificial lowering of density counts from large vessels. Green = collagen IV.

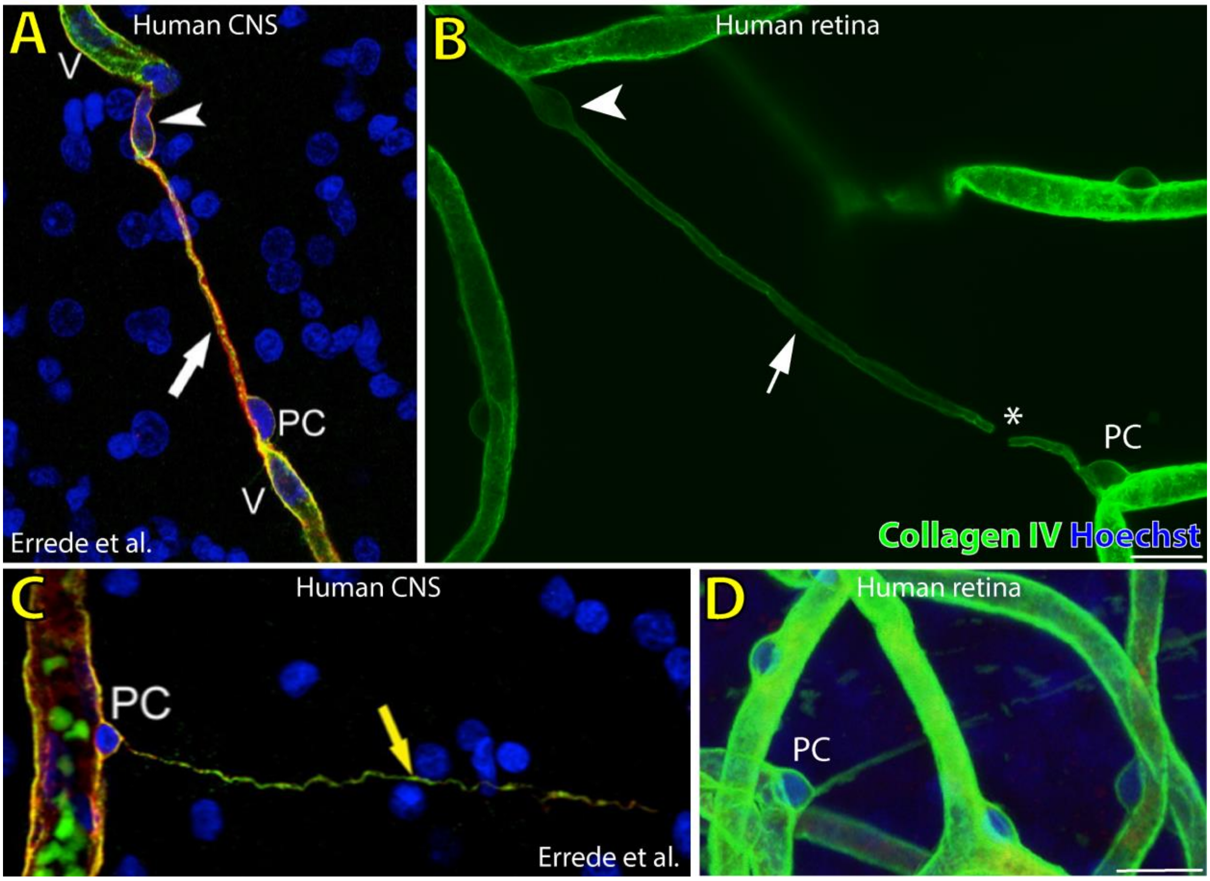

Supplementary Figure 3. Similarities between human central nervous system (CNS) and human retinal interpericyte tunnelling nanotubes (IP-TNTs). Images of human CNS IP-TNTs (A & C) are kindly reproduced from Errede et al. 2018 DOI: 10.1186/s12987-018-0114-5 (https://creativecommons.org/licenses/by/4.0/). IP-TNTs in human CNS and retina demonstrate very similar morphologies, both are very narrow tubules staining positive for collagen IV connecting pericytes (PC) attached to capillaries. Panels A and B show TNTs (white arrows) in the CNS and retina, respectively, with a similar finding of 'detached' perivascular cells (white arrowheads). Some IP-TNTs show complete breaks along their course (white asterisk; B) that may be due to post-mortem tissue change or iatrogenic change during specimen preparation. Panels C and D show IP-TNTs originating from the tip of pericyte cell bodies in human CNS and retina, respectively, that are similar in morphology. Scale bars = 20μm. Green = collagen IV; Blue = Hoechst.

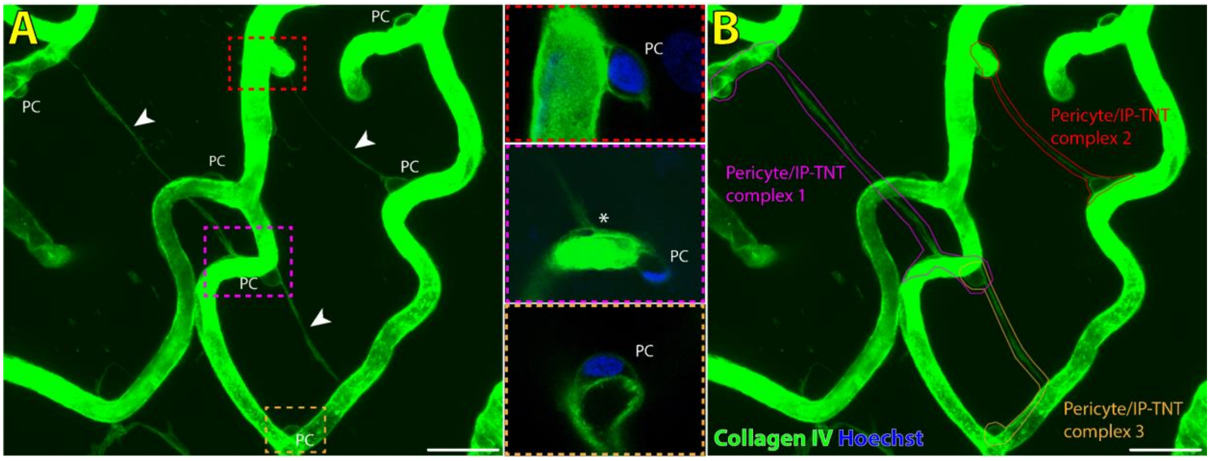

Supplementary Figure 4. Different morphological characteristics of three pericyte (PC)/interpericyte tunnelling nanotube (IP-TNT) complexes. IP-TNTs (white arrowheads) are fluorescently stained with basement membrane marker collagen IV and bridge between PCs on adjacent capillary segments (A). Dashed insets show pericytes in single optical sections of the same microphotograph in higher magnification. Note the IP-TNT visible in the magenta inset appears to terminate at distal pericyte processes (white asterisk) which communicate with the PC cell body on the opposite side of the vessel. The morphology of each pericyte/IP-TNT complex is outlined in panel B. Complex 1 (magenta outline) and complex 3 (orange outline) show IP-TNTs that terminate at vessel walls and likely communicate with distal pericyte processes further down the capillary. Complex 2 (red outline) shows an IP-TNT that directly interfaces with two PC cell bodies. Scale bars = 30µm. Green = collagen IV; Blue = Hoechst.

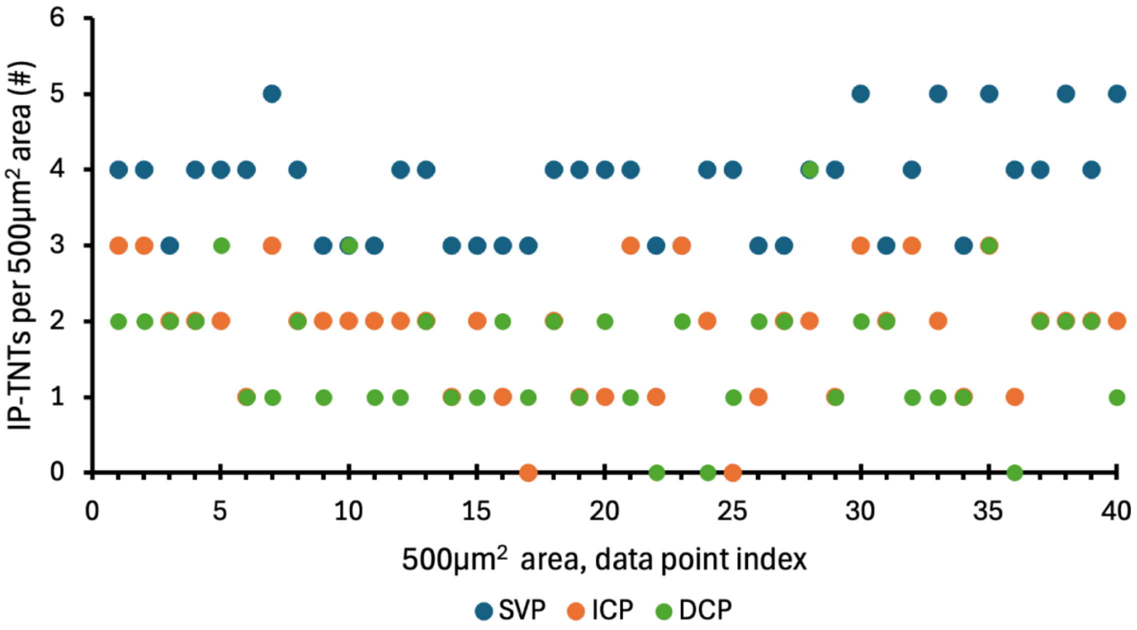

Supplementary Figure 5. Distribution of IP-TNT counts in each retinal vascular plexus per 500µm² macular area from 10 human retinal specimens.

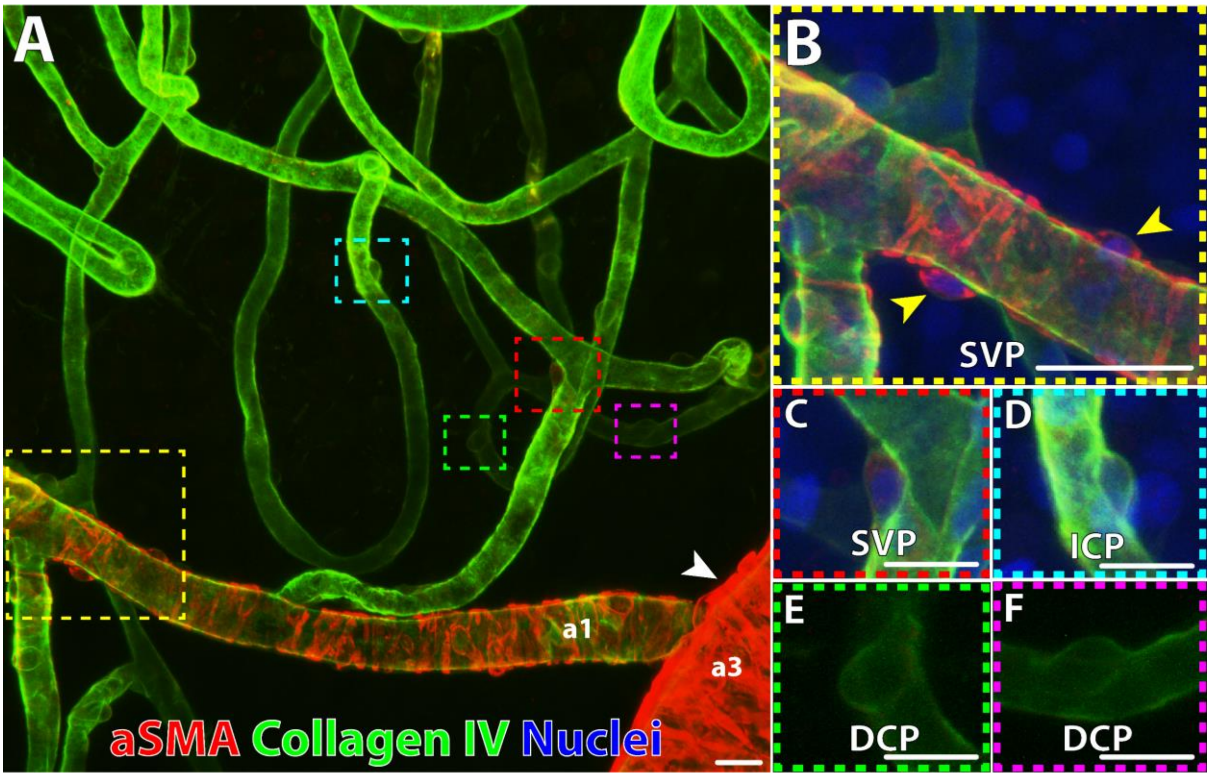

Supplementary Figure 6. Pericyte alpha smooth muscle actin (αSMA) expression in control human perifoveal arteriole and capillaries. αSMA is expressed in retinal arterioles in the form of both vascular smooth muscle cells (A; white arrow) and pericytes (Inset B and C). Pericytes of the superficial vascular plexus (SVP) are more likely to express αSMA in low order arterioles (Inset B; yellow arrows) than capillaries (Inset C). Pericytes of the intermediate and deep capillary plexus (ICP; DCP) express minimal αSMA or do not express αSMA relative to pericytes of the SVP (Inset D, E and F). Scale bars = 5 µm. Reproduced with permission from Hein et al. 2024, doi.org/10.1111/ceo.14363. Green = collagen IV; Blue = Hoechst; Red = αSMA.

Supplementary Video 1. Interpericyte tunnelling nanotube (IP-TNT, white arrowhead) in the normal human macula bridging between two pericytes (PC) located on capillaries of the superficial vascular plexus (SVP) and deep capillary plexus (DCP), bypassing the intermediate capillary plexus (ICP). Green = collagen IV; Blue = Hoechst.
